# 6-Furopyridine Hexamethylene Amiloride Is a Non-Selective P2X7 Receptor Antagonist

**DOI:** 10.3390/biom12091309

**Published:** 2022-09-16

**Authors:** Peter Cuthbertson, Amal Elhage, Dena Al-Rifai, Reece A. Sophocleous, Ross J. Turner, Ashraf Aboelela, Hiwa Majed, Richard S. Bujaroski, Iman Jalilian, Michael J. Kelso, Debbie Watson, Benjamin J. Buckley, Ronald Sluyter

**Affiliations:** 1 Illawarra Health and Medical Research Institute, Wollongong, NSW 2522, Australia; 2 Molecular Horizons and School of Chemistry and Molecular Bioscience, University of Wollongong, Wollongong, NSW 2522, Australia; 3 Department of Pharmaceutical Chemistry, Faculty of Pharmacy, Sphinx University, Assiut 71515, Egypt; 4 Monash Institute of Pharmaceutical Science (ATMCF), Monash University, Parkville, VIC 3052, Australia

**Keywords:** P2X7 receptor, P2X4 receptor, P2Y receptor, purinergic signalling, amiloride, T cell, B cell, monocyte, interleukin-1β, low affinity IgE receptor

## Abstract

P2X7 is an extracellular adenosine 5′-triphopshate (ATP)-gated cation channel present on leukocytes, where its activation induces pro-inflammatory cytokine release and ectodomain shedding of cell surface molecules. Human P2X7 can be partially inhibited by amiloride and its derivatives at micromolar concentrations. This study aimed to screen a library of compounds derived from amiloride or its derivative 5-(*N*,*N*-hexamethylene) amiloride (HMA) to identify a potential P2X7 antagonist. 6-Furopyridine HMA (6-FPHMA) was identified as a novel P2X7 antagonist and was characterized further. 6-FPHMA impaired ATP-induced dye uptake into human RPMI8226 multiple myeloma cells and human P2X7-HEK293 cells, in a concentration-dependent, non-competitive manner. Likewise, 6-FPHMA blocked ATP-induced Ca^2+^ fluxes in human P2X7-HEK293 cells in a concentration-dependent, non-competitive manner. 6-FPHMA inhibited ATP-induced dye uptake into human T cells, and interleukin-1β release within human blood and CD23 shedding from RPMI8226 cells. 6-FPHMA also impaired ATP-induced dye uptake into murine P2X7- and canine P2X7-HEK293 cells. However, 6-FPHMA impaired ATP-induced Ca^2+^ fluxes in human P2X4-HEK293 cells and non-transfected HEK293 cells, which express native P2Y_1_, P2Y_2_ and P2Y_4_. In conclusion, 6-FPHMA inhibits P2X7 from multiple species. Its poor selectivity excludes its use as a specific P2X7 antagonist, but further study of amiloride derivatives as P2 receptor antagonists is warranted.

## 1. Introduction

Purinergic signalling has multiple physiological roles [1] and comprises a range of cell surface receptors including P2X and P2Y receptors, which are typically activated by extracellular adenosine 5′-triphosphate (ATP) [2]. P2X7 is found on multiple cell types, including leukocytes [3]. ATP-induced activation of P2X7 results in the flux of Ca^2+^, Na^+^ and K^+^ cations, as well as the transfer of organic cations, including fluorescent dyes, across the cell membrane [4]. Activation of P2X7 leads to multiple pro-inflammatory downstream events. This includes activation of the NOD-like receptor family pyrin domain containing 3 (NLRP3) inflammasome, and the subsequent release of the pro-inflammatory cytokine interleukin (IL)-1β [5], and the ectodomain shedding of cell surface molecules such as the low affinity IgE receptor CD23 [6]. As such, P2X7 is a potential therapeutic target for multiple inflammatory diseases [7]. Novel compounds that block P2X7 could be used as therapeutics in inflammatory diseases or as tools to better understand the roles of P2X7 at molecular and cellular levels.

Amiloride, a commonly used K^+^-sparing diuretic that inhibits the epithelial sodium channel [8,9], and its derivatives were first shown to inhibit human and murine P2X7 three decades ago. Amiloride inhibited ATP-mediated Na^+^ fluxes in human leukaemic lymphocytes by 50% at 1 mM, while 5-(*N*,*N*-hexamethylene) amiloride (HMA) and 5-(*N*-ethyl-*N*-isopropyl) amiloride (EIPA) inhibited ATP-induced Na^+^ fluxes by 95% and 72% at 40 μM, respectively [10]. Likewise, HMA also inhibited ATP-induced ethidium^+^ uptake in human leukemic B cells by 50% at 20 μM, but concentrations > 100 μM were required for complete inhibition [11,12]. Another group demonstrated that HMA inhibited 2′(3′)-O-(4-benzoylbenzoyl) ATP (BzATP)-induced inward currents in human embryonic kidney (HEK)293 cells stably transfected with human (h) P2X7 (HEK-hP2X7 cells) by 30% at 10 μM [13]. Similarly, 40 μM HMA partially inhibited ATP- and BzATP-induced currents in *Xenopus* oocytes injected with mouse (m) P2X7 mRNA [14]. Species comparisons have revealed that HMA inhibits BzATP-induced uptake with reduced potency against mP2X7 compared to hP2X7 expressed in HEK293 cells [15]. This study also revealed that HMA potentiated mP2X7-mediated dye uptake at a relatively low concentration (16 μM) of BzATP, an effect not observed for hP2X7 [15]. Despite the inhibitory effects of amiloride and HMA against P2X7 reported above, to the best of our knowledge these compounds or other derivatives have not been pursued as P2X7 antagonists since these studies.

A library of amiloride and HMA derivatives has recently been described as potential inhibitors of human urokinase plasminogen activator (uPA) or as dual inhibitors of uPA and sodium-hydrogen exchanger isoform-1 (NHE1) [16,17,18]. It was hypothesized that this library may also contain potential P2X7 antagonists. Therefore, eighty of these compounds, including amiloride and HMA, were screened as potential novel human P2X7 antagonists with 6-furopyridine HMA (6-FPHMA) shown to inhibit hP2X7. This compound impaired hP2X7 in a non-competitive manner and blocked events downstream of P2X7 activation in human leukocytes. 6-FPHMA inhibited canine (c) P2X7 with similar potency to that for hP2X7 but was ten-fold less potent against mP2X7. However, this compound also inhibited hP2X4 and hP2Y receptors in a concentration-dependent manner with similar potency to hP2X7. As such, its poor selectivity excludes the use of 6-FPHMA as a specific P2X7 antagonist, but the future study of amiloride derivatives as single or dual P2 receptor antagonists is warranted.

## 2. Materials and Methods

### 2.1. Materials

*D*-glucose, ethidium bromide, 4-(2-hydroxyethyl)-1-piperazineethanesulfonic acid (HEPES), KCl and NaCl were from Amresco (Solon, OH, USA). Vacutainer Plus lithium heparin tubes and anti-human monoclonal antibodies (mAb), allophycocyanin-conjugated anti-CD3 (clone UCHT1), R-phycoerythrin (PE)-conjugated anti-CD4 (clone SK3) and PE-Cy7-conjugated anti-CD8 (clone RPA-T8) were from BD Biosciences (San Diego, CA, USA). Zombie near-infrared (NIR) dye was from BioLegend (San Diego, CA, USA). Foetal calf serum (FCS) was from Bovogen Biologicals (East Keilor, Australia) and heat inactivated (56 °C, 30 min) before use. Ficoll-Paque PLUS was from GE Healthcare (Uppsala, Sweden). Amiloride, ampicillin, ATP, Brilliant Blue G (BBG), CaCl_2_, dimethyl sulphoxide (DMSO), kanamycin, lipopolysaccharide (LPS) (*Escherichia coli* serotype 055:B5), Luria-Bertani (LB) agar, LB broth, MgCl_2_ and pluronic F-127 were from Sigma-Aldrich (St. Louis, MO, USA). Dulbecco’s modified phosphate-buffered saline (D-PBS), Dulbecco’s Modified Eagle Medium: Nutrient Mixture F12 (DMEM/F12), fura-2-acetoxymethyl ester (Fura-2AM), geneticin, GlutaMAX, Lipofectamine 3000, Opti-MEM, penicillin-streptomycin, RPMI-1640 medium, YO-PRO-1 iodide and PE-conjugated anti-human CD23 mAb (clone EBVCS2) were from Thermo Fisher Scientific (Waltham, MA, USA). Pyridoxalphosphate-6-azophenyl-2′,4′-disulfonic acid (PPADS) and JNJ-47965567 were from Tocris Biosciences (Minneapolis, MN, USA). 7-Aminoactinomycin (7AAD) was from Cayman Chemical (Ann Arbor, MI, USA). Amiloride derivatives, including HMA, were synthesized as previously described [16,17].

### 2.2. Cell Lines

RPMI8226 cells (European Collection of Authenticated Cell Cultures, Porton Down, UK) were maintained in RPMI-1640 medium supplemented with 2 mM GlutaMAX and 10% (*v*/*v*) FCS. HEK293 cells (American Type Culture Collection, Manassas, VA, USA) and HEK-hP2X7 cells [19] (originally provided by Dr Leanne Stokes, University of East Anglia, Norwich, UK) were maintained in DMEM/F12 medium supplemented with 2 mM GlutaMAX, 10% (*v*/*v*) FCS, 100 U/mL penicillin and 100 μg/mL streptomycin. Medium for HEK-hP2X7 cells also contained 400 μg/mL geneticin. All cell lines were maintained in 75 cm^2^ flasks at 37 °C 95% air/5% CO_2_. Cell lines regularly tested negative for mycoplasma using the MycoAlert Test Kit (Lonza, Basel, Switzerland).

### 2.3. Expression Constructs

m*P2rx7* in pPP89 (provided by Dr Leanne Stokes) was prepared as described [19]. c*P2RX7* cDNA (from an English Springer Spaniel) was cloned into an Ac-green fluorescent protein (GFP)-N1 plasmid as described [20] except using *Nhe*1/*Sac*II insertion sites. h*P2RX4* in p-Emerald (Em)GFP-N3 was prepared as described [21].

Plasmid DNA used for all transfections was transformed into competent DH5α cells (Thermo Fisher Scientific), with positive colonies selected on LB agar plates containing 100 μg/mL ampicillin (m*P2rx7*) or 50 μg/mL kanamycin (c*P2RX7* and h*P2RX4*) and single colonies were expanded in LB broth with the relevant antibiotic (37 °C, overnight). Plasmid DNA was isolated using the Wizard Plus SV Minipreps DNA Purification kit (Promega, Madison, WI, USA) according to the manufacturer’s instructions.

### 2.4. Transfection

HEK293 cells were plated at 3.5 × 10^5^ cells/well in 2 mL of complete DMEM/F12 medium and incubated (37 °C, 95% air/5% CO_2_, overnight). Cells were transfected with mP2X7-GFP (HEK-mP2X7), cP2X7-GFP (HEK-cP2X7) or hP2X4-EmGFP (HEK-hP2X4) plasmids, or mock transfected with an equivalent volume of sterile Milli-Q water (Merck Millipore, Burlington, NJ, USA), using Lipofectamine 3000 according to the manufacturer’s instructions. Cells were used for assays 48 h post-transfection. To determine transfection efficiency, cells were washed twice with D-PBS and data collected with an LSR Fortessa X-20 flow cytometer (BD Biosciences). The proportion of GFP positive cells was determined using FlowJo software v8.7.1 (BD Biosciences).

### 2.5. Plate-Based Flow Cytometric Dye Uptake for Compound Screening

RPMI8226 cells were washed thrice with low-divalent medium (LDM) (145 mM NaCl, 2 mM KCl, 0.2 mM CaCl_2_, 13 mM *D*-glucose and 10 mM HEPES, pH 7.5) and 1 × 10^5^ cells were added to each well of a 96-well flat-bottomed plate. Amiloride derivatives, at 10 μM (final concentration 5 μM) or vehicle (DMSO) were added, and plates pre-incubated (37 °C, 15 min). LDM, containing YO-PRO-1 iodide (basal) or both YO-PRO-1 iodide and ATP (250 μM, approximate EC_50_ in this assay), was added and incubated (37 °C, 8 min) before the addition of ice-cold Mg^2+^ stop solution (145 mM NaCl, 2 mM KCl, 0.2 mM CaCl_2_, 20 mM MgCl_2_ and 10 mM HEPES, pH 7.5) to halt ATP-induced dye uptake. Wells were washed twice with LDM (300× *g*, 5 min) and cells were resuspended in LDM. Data was acquired with an Attune NxT Flow Cytometer an Autosampler (Thermo Fisher Scientific). Mean fluorescent intensity (MFI) of YO-PRO-1^+^ uptake was determined using FlowJo. The respective basal fluorescence was subtracted from each ATP sample to calculate ATP-induced uptake and samples were normalized to the maximal ATP-induced response in the presence of DMSO.

### 2.6. Flow Cytometric Uptake Assays for Characterization

ATP-mediated uptake assays were conducted as described with either ethidium bromide [22] or YO-PRO-1 iodide [23]. Briefly, RPMI8226, HEK-hP2X7, HEK-mP2X7, HEK-cP2X7 or cryopreserved human peripheral blood mononuclear cells (PBMCs) [24] were washed thrice with LDM (300× *g*, 5 min), resuspended in LDM and 1 × 10^6^ cells were added to individual tubes. Cells were pre-incubated in the absence of compound (37 °C, 5 min) or in the presence of compound or vehicle (DMSO) (37 °C, 15 min) as indicated. Cells were then incubated with either ethidium bromide (25 μM) or YO-PRO-1 iodide (1 μM) in the presence of ATP or an equivalent volume of LDM (basal) (37 °C, 5 min). An equal volume of ice-cold Mg^2+^ stop solution was added and cells centrifuged (300× *g*, 5 min). Cells were washed twice in LDM (300× *g*, 5 min), resuspended and data were collected with an LSR Fortessa X-20 flow cytometer. MFI of ethidium^+^ or YO-PRO-1^2+^ was determined using FlowJo. The respective basal fluorescence was subtracted from each ATP sample to calculate ATP-induced uptake and samples were normalized to the maximal ATP-induced response in the presence of DMSO.

### 2.7. Fura-2AM Ca^2+^ Response Assay

Ca^2+^ response assays were conducted as described [21]. Briefly, HEK293, HEK-hP2X7 or HEK-hP2X4 cells were harvested and plated in black, clear-bottom 96-well plates at 5 × 10^4^ cells/well overnight (37 °C, 95% air/5% CO_2_). Cells were washed with extracellular Ca^2+^ solution (ECS) (145 mM NaCl, 2 mM CaCl_2_, 5 mM KCl, 13 mM glucose and 10 mM HEPES, pH 7.4) and incubated with Fura-2AM loading buffer (2.5 μM Fura-2AM and 0.2% pluronic acid in ECS) (37 °C, 95% air/5% CO_2_, 30 min, dark). Loading buffer was removed; cells were washed once with ECS and incubated in ECS (37 °C, 95% air/5% CO_2_, 20 min, dark) to allow Fura-2AM de-esterification. Where indicated, cells were pre-incubated with compound or vehicle (DMSO) (37 °C, 30 min, dark). Fura-2 fluorescence at 510 nm was measured every 5 s using a FlexStation 3 (Molecular Devices, San Jose, CA, USA). Baseline recordings were taken for 15 or 30 s and ATP or ECS (basal) were added using the built-in fluidics. Data was acquired using SoftMax Pro version 7.0 (Molecular Devices). Relative changes in intracellular Ca^2+^ were calculated using the ratio of Fura-2AM fluorescence at 510 nm following excitation at 340 nm and 380 nm (F_340nm/380nm_). Ca^2+^ responses were normalised to the baseline recordings using the formula ΔCa^2+^ = ΔF/F_rest_ = (F − F_rest_)/F_rest_, where F is the F_340nm/380nm_ ratio in a well at a particular time point and F_rest_ is the mean fluorescence of a well from 0–15 s or 0–30 s (prior to ATP or ECS addition) [25]. Ca^2+^ responses over time were plotted in Prism software v8.4.2 (GraphPad Software, La Jolla, CA, USA). To determine relative P2X-mediated Ca^2+^ responses, the area under the curve from 100 to 180 s was calculated with GraphPad Prism. To determine P2Y-mediated Ca^2+^ responses the area under the curve from 30 to 100 s was calculated. In both cases, ATP-induced responses were calculated by subtracting the corresponding basal response. These responses were normalised to the maximal ATP-induced response within their respective experiment.

### 2.8. Whole Blood IL-1β Release Assay

Whole blood IL-1β release assays were conducted as described [26]. Briefly, whole human blood was mixed with an equal volume of serum-free RPMI-1640 medium containing LPS (200 ng/mL), added to a flat-bottom 96-well plate and incubated (37 °C, 95% air/5% CO_2_, 105 min). Compounds (30 μM) or vehicle (DMSO) were added to respective wells and incubated (37 °C, 95% air/5% CO_2_, 15 min). ATP (6 mM) or an equivalent volume of LDM (basal) was added and plates incubated (37 °C, 95% air/5% CO_2_, 30 min). Plates were centrifuged (700× *g*, 10 min) with the resulting supernatants collected and stored at −80 °C. hIL-1β concentrations were determined using a Human IL-1β ELISA (Thermo Fisher Scientific) according to the manufacturer’s instructions. Samples above the detection limit (7500 pg/mL) were set at 7500 pg/mL for statistical analysis.

### 2.9. CD23 Shedding Assay

CD23 shedding from RPMI8226 cells was conducted as described [27], except using LDM. Briefly, 1 × 10^6^ cells in LDM were added to individual tubes and pre-incubated in the absence or presence of compound (or vehicle) (37 °C, 15 min). ATP or LDM (basal) was added, cells were incubated (37 °C, 1 min), ice-cold Mg^2+^ stop solution was added and tubes centrifuged (300× *g*, 5 min) to halt ATP-induced CD23 shedding. Cells were stained in the absence or presence of anti-CD23 mAb with 7AAD (on ice, 30 min, dark), washed once with D-PBS and data were collected using an LSR Fortessa X-20 flow cytometer. CD23 MFI on live cells was determined using FlowJo, with CD23 expression defined as the difference in MFI between cells stained in the presence or absence of anti-CD23 mAb. ATP-induced shedding was normalised to the maximal ATP-induced response in each experiment.

### 2.10. Statistical Analysis

Data are presented as mean ± standard error of the mean (SEM). All statistical analyses, graphing and curve fitting were conducted using GraphPad Prism. Where appropriate, data sets were tested for normality (Shapiro–Wilk normality test) and statistical differences were determined using a one-way ANOVA. Agonist responses were fitted to a ‘log(agonist) vs. normalized response—variable slope’ model and antagonist responses were fitted to a ‘log(inhibitor) vs. normalized response—variable slope’ model to determine the half-maximal effective concentration (EC_50_) and half-maximal inhibitory concentration (IC_50_), respectively.

## 3. Results

### 3.1. 6-FPHMA Is a Novel P2X7 Antagonist

**Figure 1:**
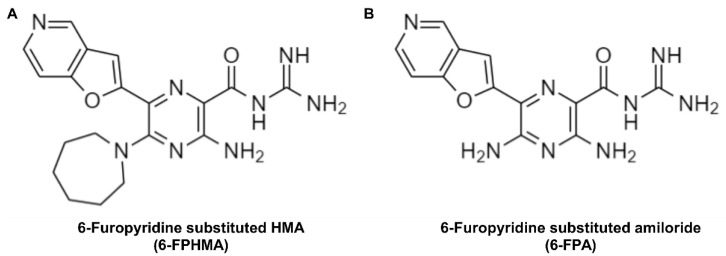
Chemical structures of (**A**) 6-furopyridine-substituted 5-(*N*,*N*-hexamethylene) amiloride (HMA) (6-FPHMA) and (**B**) 6-furopyridine-substituted amiloride (6-FPA). Structures were drawn with ChemDraw software v22.5.3 (PerkinElmer Informatics, Waltham, MA, USA).

Eighty compounds from a library of amiloride and HMA derivatives [16,17], including the parent compounds, were screened using a plate-based YO-PRO-1^2+^ dye uptake assay. A total of seven compounds were found to impair ATP-induced dye uptake by >25% (Figure S1, Supplementary Materials). Based on its chemical characteristics 6-FPHMA (Figure 1A) was identified as a possible P2X7 antagonist and selected for further characterization. The corresponding amiloride analogue, 6-furopyridine-substituted amiloride (6-FPA) (Figure 1B), was examined as a matched inactive control.

### 3.2. 6-FPHMA, but Not 6-FPA, Inhibits hP2X7-Mediated Dye Uptake

**Figure 2:**
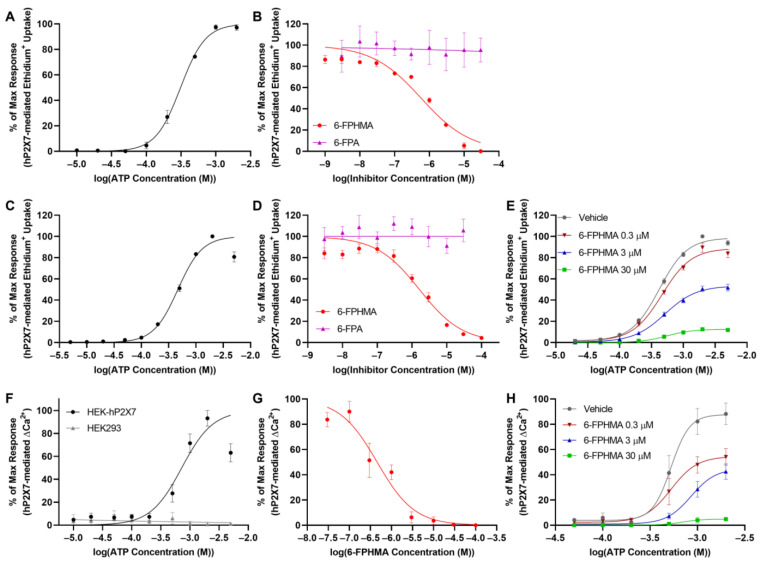
6-FPHMA inhibits human (h) P2X7-mediated dye uptake and Ca^2+^ responses in a non-competitive manner. (**A**,**B**) RPMI8226 or (**C**–**E**) human embryonic kidney (HEK)293 cells stably transfected with hP2X7 (HEK-hP2X7) were pre-incubated (**A**,**C**) alone (5 min) or (**B**,**D**,**E**) with 6-FPHMA, 6-FPA or dimethyl sulfoxide (DMSO) (vehicle) at the concentrations indicated (15 min). Cells were incubated with ethidium^+^ in the absence (basal) or presence of adenosine 5′-triphosphate (ATP) at (**A**,**C**,**E**) the concentrations indicated, (**B**) 300 μM or (**D**) 450 μM (5 min). (**A**–**E**) Ethidium^+^ uptake was assessed by flow cytometry and data normalised to the maximum ATP response in each experiment. (**F**) HEK293 or (**F**–**H**) HEK-hP2X7 cells, pre-loaded with fura-2-acetoxymethyl ester (Fura-2AM), were pre-incubated (**F**) alone (20 min) or (**G**,**H**) with 6-FPHMA or DMSO (vehicle) at the concentrations indicated (30 min). Cells were incubated in the absence (basal) or presence of ATP at (**F**,**H**) the concentrations indicated or (**G**) 720 μM. (**F**–**H**) Ca^2+^ response traces were normalised to baseline (0–15 s) and the area under the curve from 100 to 180 s was used as a measure of hP2X7-mediated Ca^2+^ flux. Responses were normalised to maximum ATP response in each experiment. (**A**–**H**) Data presented as mean ± standard error of the mean (SEM). (**A**–**E**,**H**) *n* = 5, (**F**) *n* = 6 and (**G**) *n* = 8 independent experiments.

6-FPHMA and 6-FPA were characterized using ATP-induced ethidium^+^ uptake in human RPMI8226 multiple myeloma cells, which have repeatedly been shown to express endogenous P2X7 [27,28,29,30]. To determine the EC_50_ for ATP against endogenous hP2X7, RPMI8226 cells were incubated with increasing concentrations of ATP and ethidium^+^ uptake was determined by flow cytometry (Figure S2A, Supplementary Materials). ATP induced ethidium^+^ uptake in a concentration-dependent manner with a maximum response at 1 mM and an EC_50_ of 311 ± 21 μM (Figure 2A). To determine the inhibitory effects of each compound, RPMI8226 cells were pre-incubated with DMSO (vehicle) or increasing concentrations of 6-FPHMA or 6-FPA followed by incubation with 300 μM ATP (approximate EC_50_). 6-FPHMA inhibited hP2X7-mediated dye uptake in a concentration-dependent manner with 100% blockade at 30 μM and an IC_50_ of 0.58 ± 0.05 μM (Figure 2B). In contrast, 6-FPA did not inhibit hP2X7-mediated dye uptake at any concentration up to 30 μM (Figure 2B).

To provide additional evidence that 6-FPHMA inhibits hP2X7, ATP-mediated ethidium^+^ uptake was also examined in stably transfected HEK-hP2X7 cells [19]. To determine the EC_50_ for ATP against transfected hP2X7, HEK-hP2X7 cells were incubated with increasing concentrations of ATP and ethidium^+^ uptake was determined by flow cytometry (Figure S2B, Supplementary Materials). ATP induced ethidium^+^ uptake in a concentration-dependent manner with a maximum response at 1 mM and an EC_50_ of 466 ± 17 μM (Figure 2C). To determine the inhibitory effects of each compound, HEK-hP2X7 cells were pre-incubated with DMSO (vehicle) or increasing concentrations of 6-FPHMA or 6-FPA followed by incubation with 450 μM ATP (approximate EC_50_). 6-FPHMA inhibited hP2X7-mediated dye uptake in a concentration-dependent manner with 95% blockade at 100 μM and an IC_50_ of 1.63 ± 0.42 μM (Figure 2D). Similar to RPMI8226 cells, 6-FPA did not inhibit hP2X7-mediated dye uptake at any concentration up to 30 μM (Figure 2D).

To determine if 6-FPHMA was acting in a competitive or non-competitive manner, HEK-hP2X7 cells were pre-incubated with DMSO (vehicle) or 0.3, 3 or 30 μM of 6-FPHMA then with increasing concentrations of ATP. Typical of a non-competitive inhibitor, the maximum response decreased as the concentration of 6-FPHMA increased, with maximum responses of 100 ± 0% (vehicle), 90 ± 4% (0.3 μM 6-FPHMA), 52 ± 3% (3 μM 6-FPHMA) and 13 ± 1% (30 μM 6-FPHMA) (Figure 2E). Again, consistent with a non-competitive mode of inhibition, the EC_50_ of ATP was not significantly different (*p* = 0.18, *F* = 1.8) between groups with EC_50_ values of 419 ± 24 μM (vehicle), 464 ± 52 μM (0.3 μM 6-FPHMA), 517 ± 49 μM (3 μM 6-FPHMA) and 557 ± 47 μM (30 μM 6-FPHMA) (Figure 2E).

### 3.3. 6-FPHMA Inhibits hP2X7-Mediated Ca^2+^ Responses

hP2X7 activation by ATP results in Ca^2+^ influx, and this influx can be measured using the intracellular Ca^2+^ indicator Fura-2AM. Thus, the effect of 6-FPHMA on ATP-induced Ca^2+^ influx in HEK-hP2X7 cells was examined. To determine the EC_50_ for ATP-mediated Ca^2+^ responses in HEK-hP2X7 cells, HEK-hP2X7 and non-transfected HEK293 cells were incubated with ATP and Ca^2+^ responses measured. HEK293 cells, which express endogenous hP2Y receptors [31], had an immediate Ca^2+^ response following ATP stimulation that peaked within 5 s of ATP addition and decreased to baseline by 100 s regardless of ATP concentration (Figure S3A, Supplementary Materials). In contrast, HEK-hP2X7 cells had a sustained Ca^2+^ response lasting longer than 180 s (Figure S3B, Supplementary Materials). As such, hP2X7-mediated Ca^2+^ responses were determined by measuring the area under the curve from 100–180 s (Figure S3B, Supplementary Materials). Using these criteria, ATP-induced Ca^2+^ responses in HEK293 cells were negligible while ATP-induced Ca^2+^ responses in HEK-hP2X7 were concentration-dependent with a maximal response at 2 mM and an EC_50_ of 722 ± 76 μM (Figure 2F).

To determine if 6-FPHMA could inhibit hP2X7-mediated Ca^2+^ responses, HEK-hP2X7 cells were pre-incubated with DMSO (vehicle) or increasing concentrations of 6-FPHMA then stimulated with 720 μM ATP (approximate EC_50_). 6-FPHMA inhibited hP2X7-mediated Ca^2+^ responses in a concentration-dependent manner with 100% blockade at 30 μM and an IC_50_ of 0.46 ± 0.14 μM (Figure 2G). To confirm if 6-FPHMA was acting in a non-competitive manner, HEK-hP2X7 cells were pre-incubated with DMSO (vehicle) or 0.3, 3 or 30 μM of 6-FPHMA then stimulated with increasing concentrations of ATP. Similar to ethidium^+^ uptake, the maximum response decreased as the concentration of 6-FPHMA increased, with maximum responses of 88 ± 9% (vehicle), 54 ± 7% (0.3 μM 6-FPHMA), 43 ± 6% (3 μM 6-FPHMA) and 5 ± 1% (30 μM 6-FPHMA) (Figure 2H). Further, consistent with a non-competitive mode of inhibition the EC_50_ of ATP was not significantly different (*p* = 0.63, *F* = 0.60) between groups with EC_50_ values of 526 ± 127 μM (vehicle), 526 ± 131 μM (0.3 μM 6-FPHMA), 857 ± 317 μM (3 μM 6-FPHMA) and 691 ± 151 μM (30 μM 6-FPHMA) (Figure 2H).

### 3.4. 6-FPHMA Inhibits hP2X7-Mediated Dye Uptake in Human T Cells

**Figure 3:**
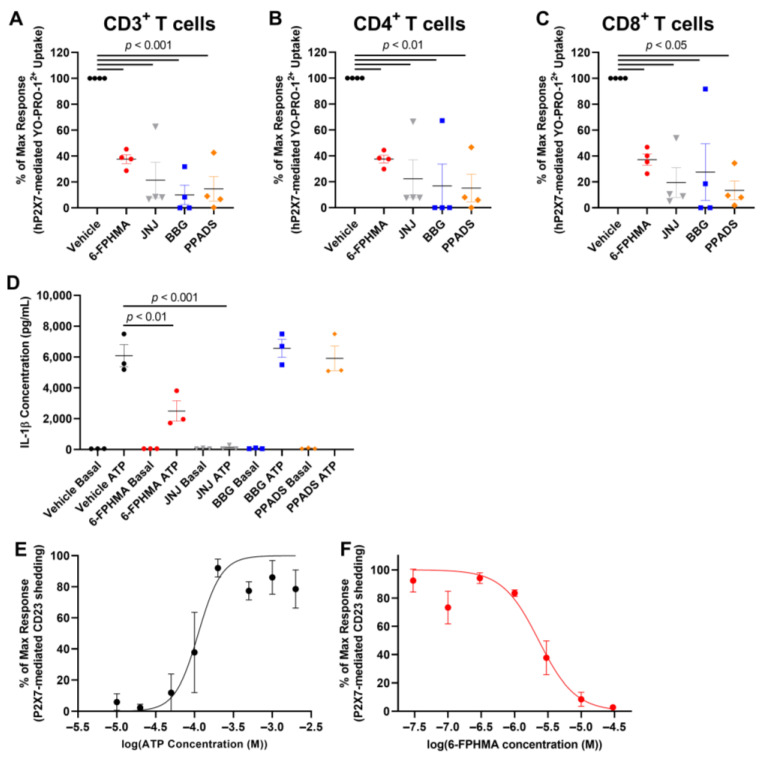
6-FPHMA inhibits P2X7 on human leukocytes. (**A**–**C**) Human peripheral blood mononuclear cells (PBMCs) were pre-incubated with DMSO (vehicle) or 30 μM 6-FPHMA, JNJ-47965567 (JNJ), Brilliant Blue G (BBG) or pyridoxalphosphate-6-azophenyl-2′,4′-disulfonic acid (PPADS) (15 min), then incubated with YO-PRO-1^2+^ in the absence (basal) or presence of 1 mM ATP (5 min). (**A**–**C**) Cells were stained with anti-CD3, anti-CD4 and anti-CD8 monoclonal antibodies (mAb) and YO-PRO-1^2+^ uptake into (**A**) CD3^+^, (**B**) CD4^+^ or (**C**) CD8^+^ T cells was assessed by flow cytometry. Data were normalised to the maximum ATP response in each experiment. (**D**) Human whole blood was incubated with RPMI-1640 medium containing lipopolysaccharide (105 min), then with DMSO (vehicle) or 30 μM 6-FPHMA, JNJ, BBG or PPADS (15 min) and finally in the absence (basal) or presence of 6 mM ATP (30 min). Interleukin (IL)-1β in cell-free supernatants was measured by ELISA. (**E**,**F**) RPMI8226 cells were pre-incubated (**E**) alone (5 min) or (**F**) with 6-FPHMA at the concentrations indicated (15 min) then incubated in the absence (basal) or presence of ATP (**E**) at the concentrations indicated or (**F**) 200 μM (1 min). (**E**,**F**) Cells were stained with anti-CD23 mAb and 7-aminoactinomycin (7AAD), CD23 shedding was determined by flow cytometry and data were normalised to the maximum ATP response in each experiment. (**A**–**F**) Data presented as mean ± SEM. (**A**–**C**) *n* = 4 and (**D**) *n* = 3 independent donors, (**E**) *n* = 3 and (**F**) *n* = 4 independent experiments. (**A**–**D**) Symbols represent individual donors.

To determine if 6-FPHMA could inhibit P2X7-mediated dye uptake into human T cells, and to examine how this compound compares to other P2X7 antagonists, human PBMCs were pre-incubated with vehicle (DMSO), 30 μM 6-FPHMA, or the well-established P2X7 antagonists, JNJ-47965567, BBG or PPADS [4], also at 30 μM, followed by 1 mM ATP, a concentration previously shown to induce YO-PRO-1^2+^ dye uptake in human PBMCs [32]. Dye uptake into human CD3^+^ T cells, and CD4^+^ or CD8^+^ T cell subsets was determined by flow cytometry (Figure S4, Supplementary Materials). 6-FPHMA reduced ATP-induced uptake into CD3^+^ T cells by 62 ± 3%, while JNJ-47965567, BBG and PPADS reduced uptake into these cells by 79 ± 14%, 90 ± 8% and 85 ± 9%, respectively (Figure 3A). Similar inhibitory responses were seen in CD4^+^ T cells, with 6-FPHMA, JNJ-47965567, BBG and PPADS reducing uptake by 62 ± 3%, 78 ± 15%, 83 ± 17% and 85 ± 11%, respectively (Figure 3B), and in CD8^+^ T cells, with 6-FPHMA, JNJ-47965567, BBG and PPADS reducing uptake by 62 ± 4%, 80 ± 11%, 72 ± 22% and 86 ± 7%, respectively (Figure 3C). All antagonists (except 6-FPHMA) showed reduced inhibition of dye uptake into T cells from the same donor compared to the other three donors.

### 3.5. 6-FPHMA Inhibits hP2X7-Mediated IL-1β Release and CD23 Shedding from Human Leukocytes

6-FPHMA inhibited hP2X7-mediated dye uptake and Ca^2+^ flux in cell lines, and reduced dye uptake in human T cells. Therefore, the effect of 6-FPHMA on P2X7-mediated downstream events was examined. P2X7 activation leads to activation of the NLRP3 inflammasome and the release of IL-1β [33]. To determine the effect of 6-FPHMA, and compare its inhibitory action to other P2X7 antagonists, ATP-induced IL-1β release in human blood [26] was examined. In the absence of ATP (basal), IL-1β release in blood was negligible (<100 pg/mL) regardless of vehicle or compound (Figure 3D). 6-FPHMA and JNJ-47965567 reduced ATP-induced IL-1β by 56 ± 15% and 98 ± 1%, respectively, compared to the vehicle control (Figure 3D). Unexpectedly BBG and PPADS did not alter ATP-induced IL-1β release (Figure 3D).

P2X7 activation leads to the shedding of CD23 from human immune cells [6] and RPMI8226 cells have previously been used to examine such responses in this cell line model of human leukocytes [27,34]. To confirm that ATP could induce CD23 shedding, the EC_50_ of ATP for CD23 shedding from RPMI8226 cells was first determined. RPMI8226 cells were incubated with increasing concentrations of ATP and CD23 expression on live cells was determined by flow cytometry (Figure S5, Supplementary Materials). ATP induced CD23 shedding in a concentration-dependent manner with a maximal response at 200 μM and an EC_50_ of 112 ± 28 μM (Figure 3E). To determine if 6-FPHMA could inhibit P2X7-mediated CD23 shedding, RPMI8226 cells were pre-incubated with DMSO (vehicle) or increasing concentrations of 6-FPHMA followed by 200 μM ATP. 6-FPHMA inhibited ATP-induced CD23 shedding in a concentration-dependent manner with 97% blockade at 30 μM and an IC_50_ of 2.3 ± 0.5 μM (Figure 3F).

### 3.6. 6-FPHMA Inhibits mP2X7- and cP2X7-Mediated Dye Uptake

**Figure 4:**
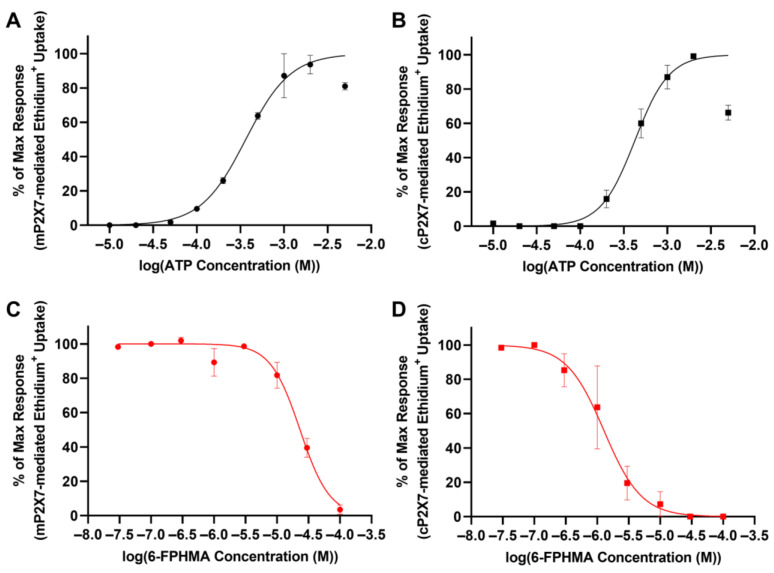
6-FPHMA inhibits murine (m) and canine (c) P2X7 in a concentration-dependent manner. HEK293 cells were transfected with (**A**,**C**) mP2X7-green fluorescent protein (GFP) or (**B**,**D**) cP2X7-GFP and pre-incubated (**A**,**B**) alone (5 min) or (**C**,**D**) with 6-FPHMA at the concentrations indicated (15 min). Cells were incubated with ethidium^+^ in the absence (basal) or presence of ATP at (**A**,**B**) the concentrations indicated, (**C**) 360 μM or (**D**) 420 μM (5 min). (**A**–**D**) Ethidium^+^ uptakes in GFP^+^ cells were assessed by flow cytometry and data normalised to the maximum ATP response in each experiment. (**A**–**D**) Data presented as mean ± SEM. (**A**–**D**) *n* = 3 independent experiments.

Common P2X7 antagonists have species-specific potencies, with many compounds having reduced inhibition against mP2X7 compared to hP2X7 [35], while cP2X7 and hP2X7 respond similarly to ATP [36,37] and a range of P2X7 antagonists [19,20,36,37,38]. As such, the ability of 6-FPHMA to inhibit mP2X7 and cP2X7 was examined. To determine the EC_50_ for ATP against mP2X7 and cP2X7, HEK293 cells were transiently transfected with mP2X7-GFP (HEK-mP2X7), cP2X7-GFP (HEK-cP2X7) or mock transfected, and ethidium^+^ uptake conducted as described above. Mock-transfected HEK293 cells showed no ATP-induced ethidium^+^ uptake (Figure S6, Supplementary Materials). ATP induced ethidium^+^ uptake into GFP^+^ HEK-mP2X7 or HEK-cP2X7 cells (Figure S6, Supplementary Materials) in a concentration-dependent manner with a maximum response at 2 mM for both mP2X7 and cP2X7 and EC_50_ values of 364 ± 48 μM (Figure 4A) or 424 ± 67 μM (Figure 4B), respectively. To determine the IC_50_ of 6-FPHMA against mP2X7 and cP2X7, transfected cells were pre-incubated with increasing concentrations of 6-FPHMA then incubated with 360 μM or 420 μM ATP (respective approximate EC_50_ values). 6-FPHMA inhibited mP2X7- or cP2X7-mediated dye uptake in a concentration-dependent manner with 96% blockade at 100 μM or 100% blockade at 30 μM, respectively, and IC_50_ values of 23 ± 2.4 μM (Figure 4C) or 1.28 ± 0.47 μM (Figure 4D), respectively.

### 3.7. 6-FPHMA Inhibits hP2X4- and hP2Y-Mediated Ca^2+^ Responses

**Figure 5:**
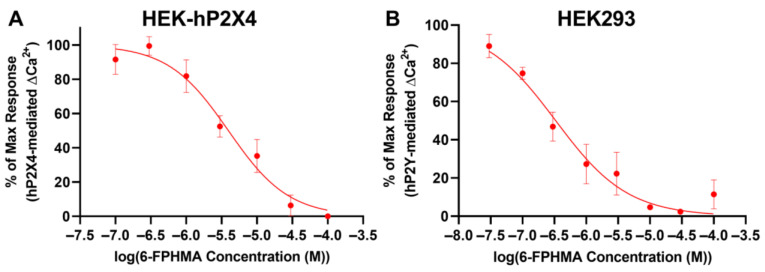
6-FPHMA inhibits hP2X4 and hP2Y receptors in a concentration-dependent manner. (**A**) HEK-hP2X4 or (**B**) HEK293 cells, pre-loaded with Fura-2AM, were pre-incubated with 6-FPHMA or DMSO (vehicle) at the concentrations indicated (30 min). Cells were stimulated, or not (basal), with ATP at (**A**) 1.0 μM or (**B**) 2.5 μM. Ca^2+^ responses were normalised to baseline (**A**: 0–15 s, **B**: 0–30 s) and the area under the curve from (**A**) 100 to 180 s or (**B**) 30 to 100 s was used as a measure of hP2X4- or hP2Y-mediated Ca^2+^ flux, respectively. Data were normalised to the maximum ATP response in each experiment. (**A**,**B**) Data presented as mean ± SEM. (**A**) *n* = 4–7 and (**B**) *n* = 4 independent experiments.

Next, to determine whether 6-FPHMA could inhibit another P2X receptor HEK293 cells were transfected with hP2X4-GFP (HEK-hP2X4). Transfection efficiency was determined by flow cytometry (Figure S7A, Supplementary Materials) before proceeding with Ca^2+^ response assays. Transiently transfected HEK-hP2X4 cells were pre-incubated with increasing concentrations of 6-FPHMA then incubated with 1 μM ATP (approximate EC_90_) as described [21]. Similar to HEK-hP2X7 cells, HEK-hP2X4 cells had a sustained response lasting longer than 180 s (Figure S7B, Supplementary Materials), so the area under the curve from 100 to 180 s was used as a measure of hP2X4-mediated Ca^2+^ flux. 6-FPHMA inhibited hP2X4 in a concentration-dependent manner with 100% inhibition at 100 μM and an IC_50_ of 3.95 ± 0.19 μM (Figure 5A).

As mentioned previously, HEK293 cells express endogenous hP2Y receptors, including hP2Y_1_, hP2Y_2_ and hP2Y_4_ [31] and activation of these receptors can induce Ca^2+^ responses in these cells [21,31]. To confirm the presence of endogenous hP2Y receptors on HEK293 cells, these cells were incubated with increasing concentrations of ATP and Ca^2+^ responses measured. ATP-induced Ca^2+^ responses were determined by measuring the area under the curve from 30 to 100 s (Figure S7C, Supplementary Materials). ATP induced Ca^2+^ in a concentration-dependent manner with a maximum response at 300 μM and an EC_50_ of 2.50 ± 1.01 μM (Figure S7D, Supplementary Materials). To determine if 6-FPHMA could block hP2Y receptors, HEK293 cells were pre-incubated with various concentrations of 6-FPHMA then incubated with 2.5 μM ATP (EC_50_) and Ca^2+^ responses were measured (Figure S7E, Supplementary Material). 6-FPHMA inhibited endogenous hP2Y-mediated Ca^2+^ responses in a concentration-dependent manner with 97% inhibition at 30 μM and an IC_50_ of 0.33 ± 0.13 μM (Figure 5B).

## 4. Discussion

6-FPHMA was identified as a P2X7 antagonist following the screening of 80 amiloride or HMA derivatives, including the parent compounds. Characterization of this novel antagonist revealed that it inhibited hP2X7 in a non-competitive manner and that it could also block events downstream of P2X7 activation. 6-FPHMA displayed species-specific potency, with similar IC_50_ values against hP2X7 and cP2X7 but it was ten-fold less active against murine P2X7, similar to that observed for other P2X7 antagonists [19,20,35,36,37,38]. The current study demonstrated that 6-FPHMA also inhibited hP2X4 and hP2Y receptors in a concentration-dependent manner with similar potency to hP2X7. Collectively, this study identified 6-FPHMA as a P2X7 antagonist, but this compound lacked selectivity, limiting its use as a specific P2X7 antagonist. Nevertheless, given this broad inhibitory action against P2 receptors, the possibility remains that other amiloride or HMA derivatives may be identified or developed as more selective P2X or P2Y antagonists.

6-FPHMA inhibited hP2X7 more potently than any other amiloride or HMA derivative described to date with IC_50_ values ranging from 0.46 μM to 2.3 μM depending on the assay and cell type used. Previous studies [10,11,12,13,14] and the screening assay in the current study indicated that amiloride blocks hP2X7 relatively poorly but derivatives with 5-alkylamino groups, like HMA and EIPA, have increased potency against this receptor. This hydrophobic 5-substitution may allow these compounds to better interact with the key residues in the previously identified allosteric P2X7 drug-binding pockets [39,40,41]. Whether amiloride, HMA or their derivatives, including 6-FPHMA, bind this pocket remains to be shown. Moreover, since 6-FPHMA was a more potent inhibitor of P2X7 than HMA, the addition of a second hydrophobic group, such as furopyridine, at the 6-position may increase the ability of these compounds to interact with the drug-binding pocket. It should be noted that the 5-hexamethylene substitution is likely to be more important than the 6-furopyridine group for interactions in the drug-binding pocket, as 6-FPA, which contains a 6-furopyridine group but not a 5-hexamethylene substitution, did not inhibit P2X7. Studying other amiloride or HMA derivatives with similar functional groups to 6-FPHMA may help elucidate the binding mechanisms of these compounds.

6-FPHMA inhibited P2X7-mediated dye uptake into human T cells, including CD4^+^ and CD8^+^ T cell subsets, and human RPMI8226 multiple myeloma cells. This parallels earlier works that identified amiloride and its analogues as P2X7 antagonists using human leukemic lymphocytes [10,12]. Furthermore, the present study demonstrated for the first time that an HMA derivative can inhibit events downstream of hP2X7 activation, namely IL-1β release and CD23 shedding. Despite reduced inhibition of P2X7 on T cells compared to BBG and PPADS, 6-FPHMA inhibited IL-1β release in human blood more potently than either BBG or PPADS. This may indicate that 6-FPHMA would be a more potent P2X7 antagonist in vivo than BBG or PPADS, although it should be noted JNJ-47965567 inhibited P2X7 better than 6-FPHMA in either assay. It remains unclear as to why BBG or PPADS lacked inhibitory activity in whole blood. However, this finding has implications for the use of such antagonists in vivo and whether they also have reduced efficacy in rodent models of disease.

The current study demonstrated, for the first time, that an amiloride derivative can inhibit P2X7 from multiple species. 6-FPHMA had similar potency against hP2X7 and cP2X7 but reduced potency against mP2X7. This may be due to the greater sequence identity between hP2X7 and cP2X7 (~85%) compared with hP2X7 and mP2X7 (~80%) [4]. Further, the decreased potency against mP2X7 compared to hP2X7 seen in the current study reflects what is observed for many other P2X7 antagonists, including PPADS, pyridoxal-5′-phosphate-6-(2′-naphthylazo-6′-nitro-4′,8′-disulfonate) tetrasodium salt (PPNDS), MRS2159 and A740003 [35]. Mapping of panda (pd) P2X7 identified a hydrophobic pocket involving five key residues (F95, F103, M105, F293 and V312) that can bind structurally distinct non-competitive P2X7 antagonists, A740003, A804598, AZ10606120, GW791343 and JNJ-47965567 [39]. Other studies indicated that the P2X7 antagonists A438079, AZ116453731, BBG, KN-62, calmidazolium and ZINC58368839 bind to this same pocket or an overlapping but distinct inter-subunit allosteric site involving additional residues including F88, D92, T94 and F108 [40,41]. Given these findings and that 6-FPHMA inhibited P2X7 in a non-competitive manner, it is likely, but not confirmed, that 6-FPHMA is binding in a similar location.

Contrary to the initial aim to identify a novel P2X7 antagonist, the current study provides the first evidence that an HMA derivative has the potential to inhibit multiple P2 receptors. 6-FPHMA inhibited hP2X7, hP2X4 and hP2Y receptors at similar potency. Further, 6-FPHMA can also inhibit both human uPA and NHE1 [17]. As mentioned above, five selective P2X7 antagonists bind non-competitively to the same allosteric hydrophobic drug-binding pocket on P2X7, but these compounds are unable to bind a similar pocket on P2X4 due to their relatively large size [39]. 6-FPHMA is larger than the smallest of these antagonists, so it is also unlikely that 6-FPHMA is binding to this hydrophobic pocket on P2X4. This suggests 6-FPHMA is binding to a different site on P2X4 to that of P2X7. Alternatively, 6-FPHMA may be binding both P2X4 and P2X7 at structurally similar allosteric drug-binding sites, such as the homologous amino acid residues I312 and I310 which are involved in the binding of the P2X4 antagonist BX430 [42]. If so, this would imply that 6-FPHMA does not bind the well-described allosteric binding pocket discussed above. However, the notion that 6-FPHMA binds structurally similar allosteric drug-binding sites on P2X4 and P2X7 does not explain the inhibition of hP2Y receptors by 6-FPHMA. This may suggest that 6-FPHMA is binding to a unique site on each P2 receptor studied, or it may indicate that 6-FPHMA is inserting directly into the cell membrane, as previously shown for the non-selective P2 receptor antagonists, PPADS and MRS2159 [43]. If true, this suggests that 6-FPHMA may have the ability to inhibit other P2 receptors. Future in vitro or in silico studies could examine where and how 6-FPHMA binds to P2 receptors. Moreover, 6-FPHMA may be suitable as a lead compound to develop a dual P2X7/P2X4 antagonist. Dual blockade of P2X7 and P2X4 may be beneficial as a therapy in a number of inflammatory diseases, such as cardiovascular disease [44,45] or neuroinflammatory disorders [46,47], where both receptors have important roles. However, concerning the development of such compounds as specific P2 receptor antagonists, the potential inhibition of uPA and/or NHE1 also needs to be considered.

## 5. Conclusions

In conclusion, this study identified and characterized a novel non-competitive P2X7 antagonist, 6-FPHMA. This compound had greater potency then either amiloride or HMA and impaired events downstream of P2X7 activation. Further, 6-FPHMA inhibited P2X7 from other species; however, it also inhibited human P2X4 and P2Y receptors, limiting its use as a P2X7 antagonist. Nevertheless, this suggests that 6-FPHMA may be a starting point for the development of more selective P2 receptor antagonists. Therefore, the further study of amiloride derivatives as P2 receptor antagonists is warranted.

## Data Availability

The data presented in this study are available on request from the corresponding author.

## Supplementary Materials

The following supporting information can be downloaded at: https://www.mdpi.com/article/10.3390/biom12091309/s1, Figure S1: Compound screening revealed 6-furopyridine substituted HMA as a candidate for further characterisation, Figure S2: Representative flow cytometric gating strategies to determine dye uptake into RPMI8226 and HEK-hP2X7 cells, Figure S3: Ca^2+^ response traces in non-transfected and P2X7-transfected HEK293 cells, Figure S4: Representative flow cytometric gating strategy to determine dye uptake into human T cells, Figure S5: Representative flow cytometric gating strategy to determine CD23 shedding from RPMI8226 cells, Figure S6: Representative flow cytometric histograms of dye uptake into HEK-mP2X7 and HEK-cP2X7 cells, Figure S7: Transfection efficiency and Ca^2+^ response traces in HEK-hP2X4 and HEK293 cells.
