# Supplementary material for: 6-Furopyridine Hexamethylene Amiloride Is a Non-Selective P2X7 Receptor Antagonist
Source: Biomolecules. 2022 Sep 16;12(9):1309. doi: 10.3390/biom12091309 (PMC9496321; doi:10.3390/biom12091309)
Supplement: Supplementary file 1 [file biomolecules-12-01309-s001.zip › biomolecules-1882691-supplementary.pdf]

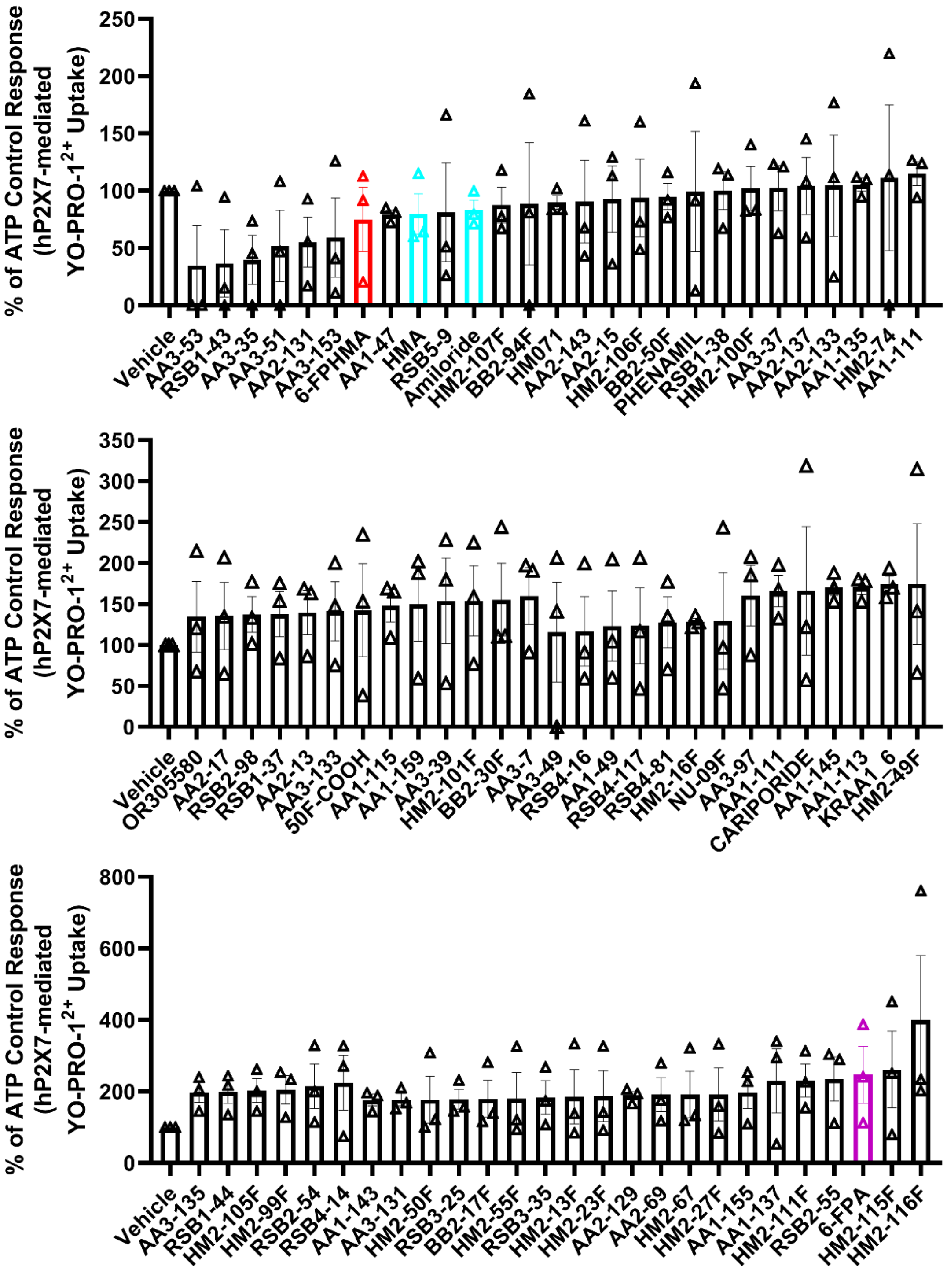

Figure S1: Compound screening revealed 6-furopyridine HMA (6-FPHMA), but not its analogue 6-furopyridine-substituted amiloride (6-FPA), as a potential P2X7 antagonist. RPMI8226 cells were plated and pre-incubated with 5 μM of each compound or vehicle (DMSO) for 15 min, then with YO-PRO-1^{2+} in the absence (basal) or presence of 250 μM ATP. YO-PRO-1^{2+} uptake was assessed by flow cytometry. Basal fluorescence was subtracted to determine the ATP-induced fluorescence for each compound and data were normalised to the vehicle ATP-induced response in each experiment. Data is presented as mean ± SEM. *n* = 3 independent experiments. Symbols represent individual experiments. Amiloride and HMA (cyan), 6-FPHMA (red) and 6-FPA (magenta) have been coloured for easier identification.

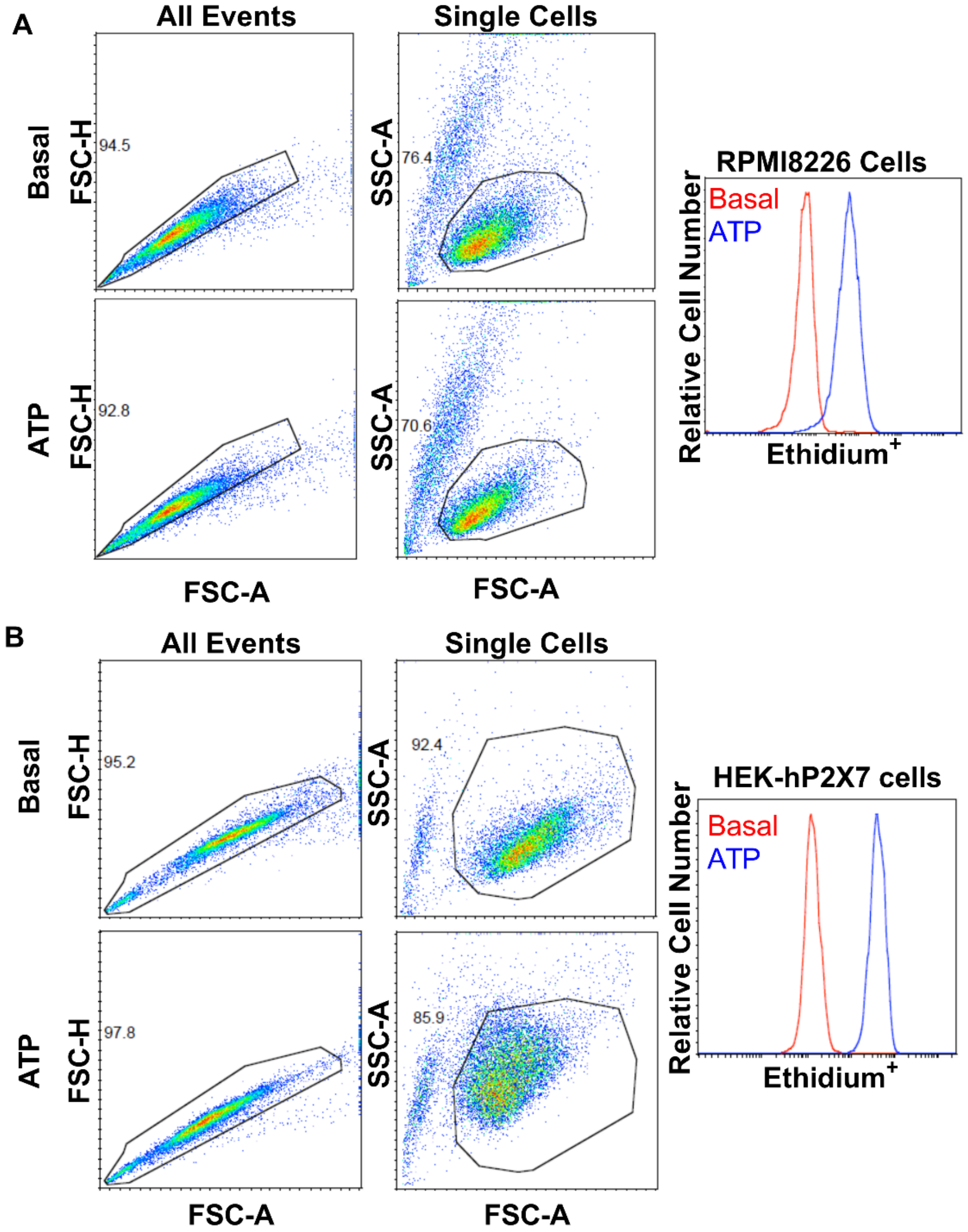

Figure S2: Representative flow cytometric gating strategies to determine dye uptake into RPMI8226 and HEK-hP2X7 cells. (**A**) RPMI8226 or (**B**) HEK-hP2X7 cells were pre-incubated (5 min) alone then incubated with ethidium^{+} in the absence (basal) or presence of 2 mM ATP (5 min). (**A, B**) Ice-cold Mg^{2+} stop solution was added and data acquired using flow cytometry. Forward scatter (FSC)-height (H) and FSC-area (A) were used to identify single cells. Side scatter (SSC)-A and FSC-A were used to identify live cells. Ethidium^{+} MFI measured in live single cells. Images are from a single experiment for each cell type, with a consistent gating strategy used for all cell line-based dye uptake experiments.

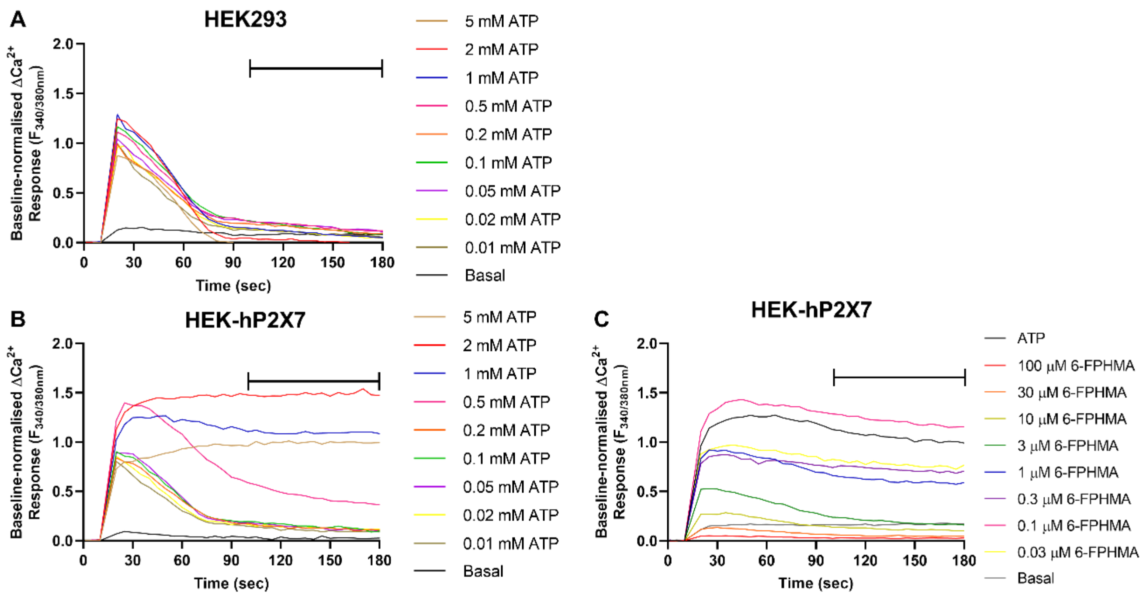

Figure S3: Ca^{2+} response traces in non-transfected and P2X7-transfected HEK293 cells. (**A**) HEK293 or (**B, C**) HEK-hP2X7 cells, pre-loaded with Fura-2AM, were pre-incubated (**A, B**) alone (20 min) or (**C**) in the presence of DMSO (vehicle) or 6-FPHMA at the concentrations indicated (30 min). Cells were incubated in the absence (basal) or presence of ATP at (**A, B**) the concentrations indicated or (**C**) 720 µM. (**A-C**) Ca^{2+} traces ($F_{340/380nm}$) were normalised to baseline (0-15 s) readings and the area under the curve from 100-180 s (bar marker) was used as a measure of hP2X7-mediated Ca^{2+} flux. (**A-C**) Data presented as mean. (**A-C**) n = 6 independent experiments.

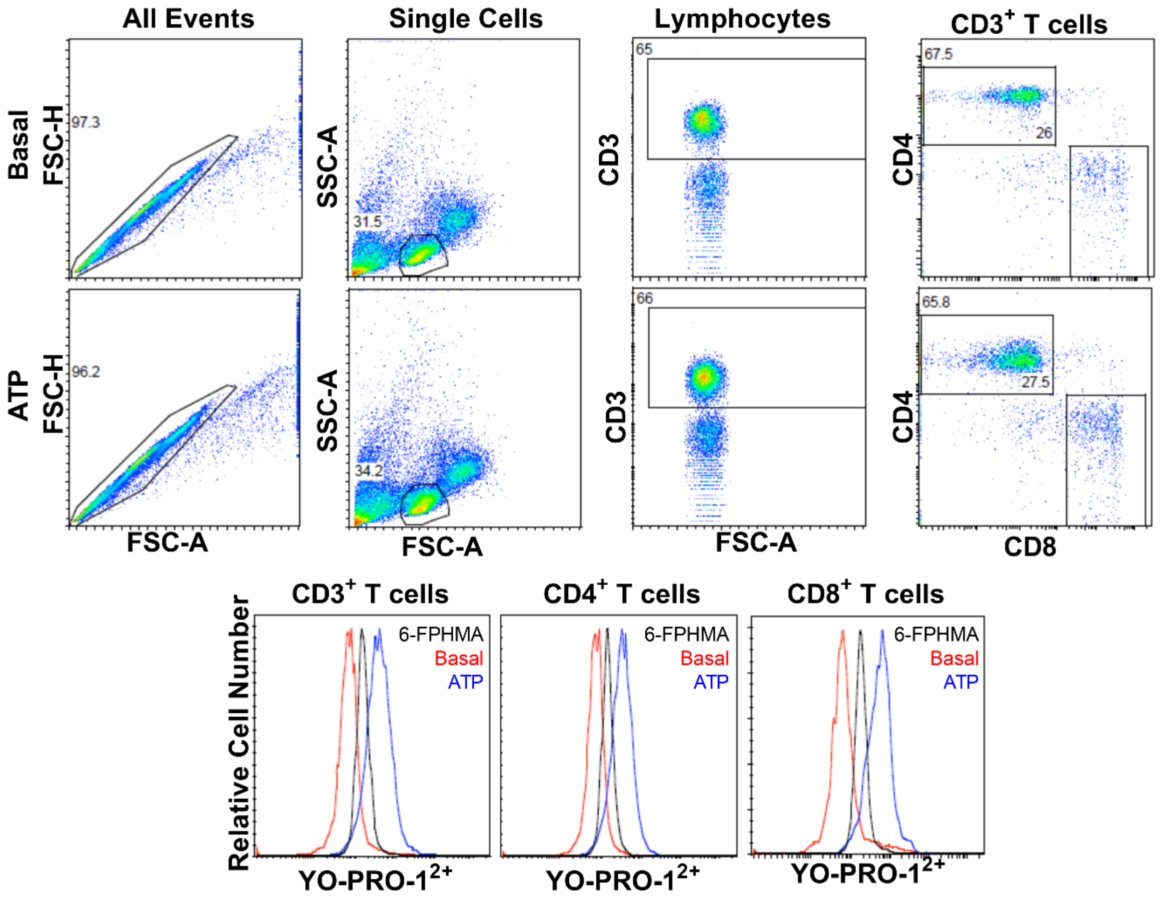

Figure S4: Representative flow cytometric gating strategy to determine dye uptake into human T cells. PBMCs were pre-incubated with 6-FPHMA (15 min) then incubated with YO-PRO-1^{2+} in the absence (basal) or presence of 1 mM ATP (5 min). Ice-cold Mg^{2+} stop solution was added and cells were stained with anti-CD3, anti-CD4 and anti-CD8 mAbs and data acquired using flow cytometry. FSC-H and FSC-A were used to identify single cells. SSC-A and FSC-A were used to identify lymphocytes. $CD3^+$ T cells were identified and divided into $CD4^+$ and $CD8^+$ subsets as shown. YO-PRO-1^{2+} MFI was measured in $CD3^+$, $CD4^+$ or $CD8^+$ T cells as shown. Images are from a single experiment, with a consistent gating strategy used for all PBMC-based dye uptake experiments.

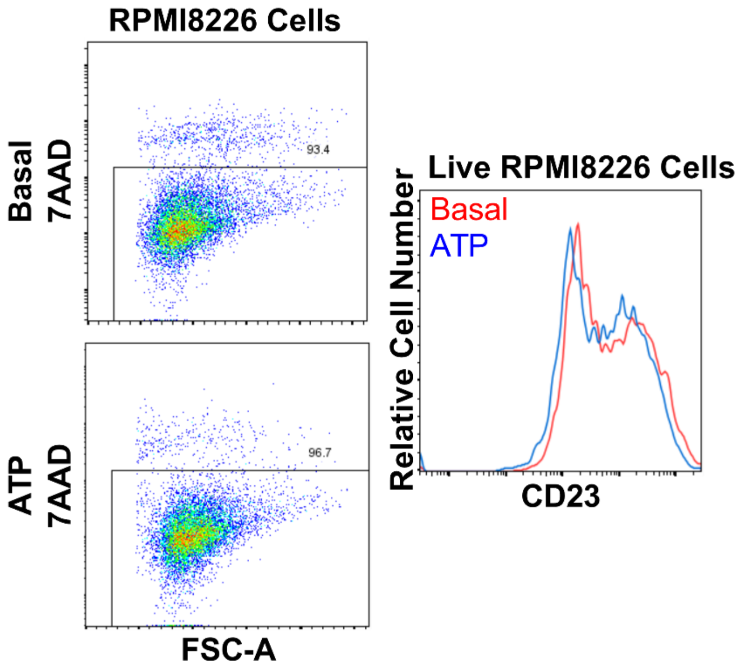

Figure S5: Representative flow cytometric gating strategy to determine CD23 shedding from RPMI8226 cells. RPMI8226 cells were pre-incubated (5 min) alone then incubated in the absence (basal) or presence of 2 mM ATP (1 min). Ice-cold Mg^{2+} stop solution was added and cells were stained with anti-CD23 mAb and the live/dead stain 7AAD. Data was acquired using flow cytometry. FSC-H, FSC-A and SSC-A were used to identify single RPMI8226 cells as shown in **Figure S2A**. FSC-A and 7AAD were used to identify live cells and CD23 MFI on live cells was determined as shown. Images are from a single experiment, with a consistent gating strategy used for all CD23 shedding experiments

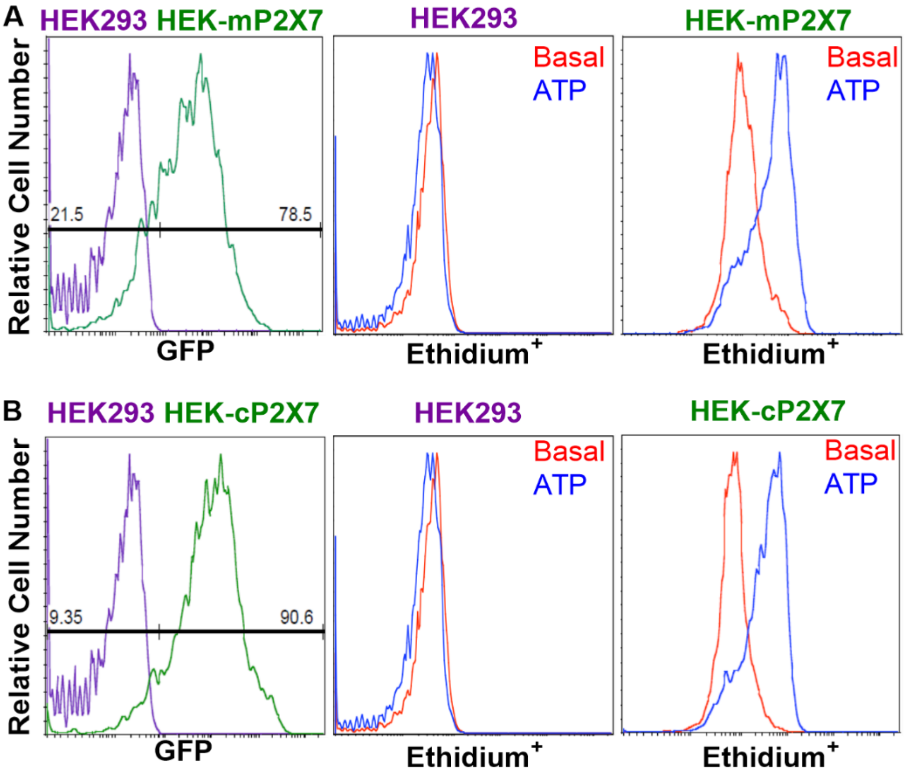

Figure S6: Representative flow cytometric histograms of dye uptake into HEK-mP2X7 and HEK-cP2X7 cells. (**A, B**) HEK293 or transfected (**A**) HEK-mP2X7 or (**B**) HEK-cP2X7 cells were pre-incubated (5 min) alone then incubated with ethidium^{+} in the absence (basal) or presence of 2 mM ATP (5 min). (**A, B**) Ice-cold Mg^{2+} stop solution was added and data acquired using flow cytometry. FSC-H, FSC-A and SSC-A were used to identify single HEK cells as shown in **Figure S2B**. GFP was used to identify transfected cells. Ethidium^{+} MFI measured in GFP^{-} (left markers) or GFP^{+} (right markers) cells as shown. Images are from a single experiment for each cell type, with a consistent gating strategy used for all HEK-mP2X7 and HEK-cP2X7 dye uptake experiments.

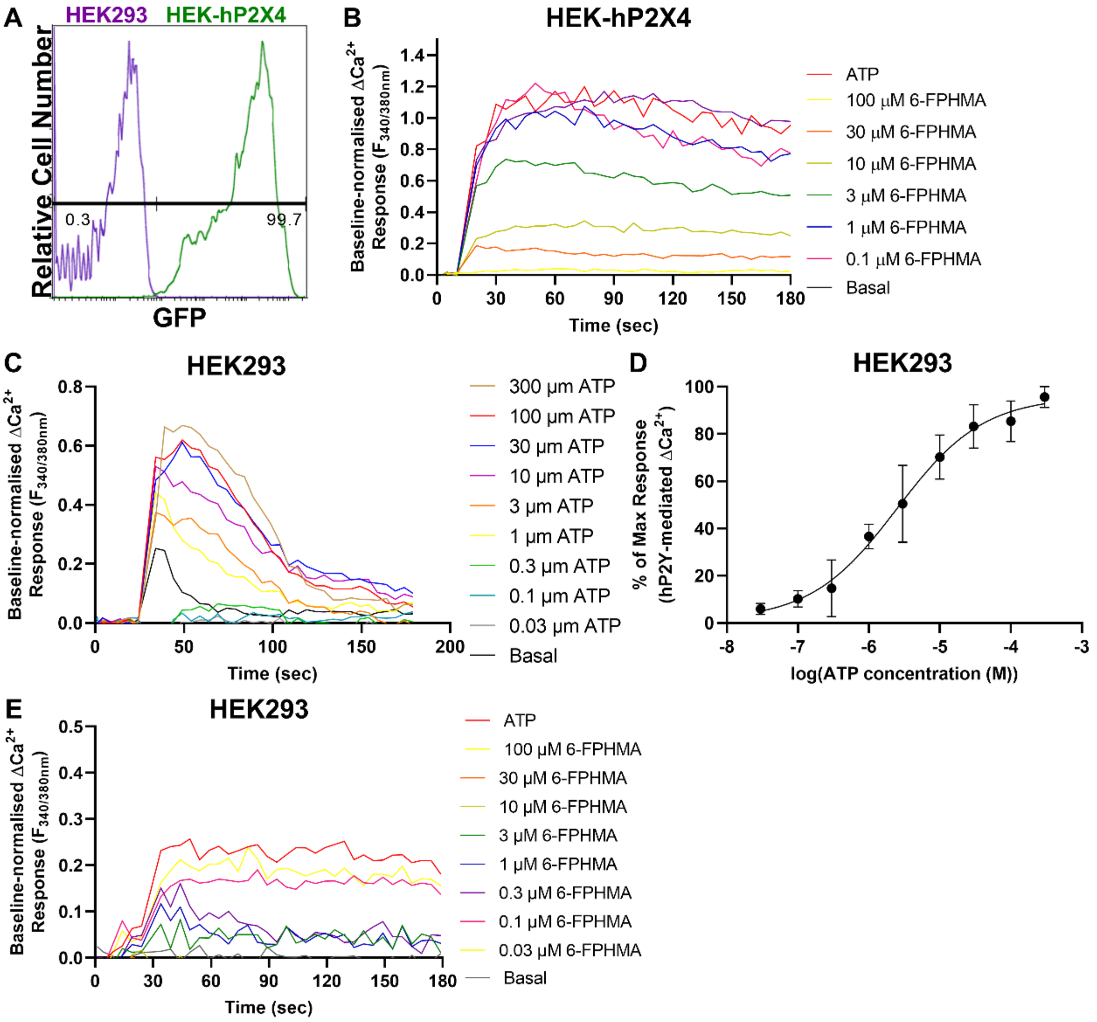

Figure S7: Transfection efficiency and Ca^{2+} response traces in HEK-hP2X4 and HEK293 cells. (**A**) HEK293 or HEK-hP2X4 cells were examined by flow cytometry to determine transfection efficiency. FSC-H, FSC-A and SSC-A were used to identify single HEK293 cells as shown in **Figure S2B** and GFP^- (left marker) and GFP^+ (right marker) cells were identified. Image is from a single experiment, with a consistent gating strategy used to check transfection efficiency for all HEK-hP2X4 experiments. (**B**) HEK-hP2X4 or (**C-E**) HEK293 cells, pre-loaded with Fura-2AM, were pre-incubated (**C, D**) alone (20 min) or (**B, E**) in the presence of DMSO (vehicle) or 6-FPHMA at the concentrations indicated (30 min). Cells were incubated in the absence (basal) or presence of ATP at (**B**) 1 µM, (**C, D**) the concentrations indicated or (**E**) 2.5 µM. (**B, C, E**) Ca^{2+} traces ($F_{340/380nm}$) were normalised to baseline (**B**: 0-15 s, **C, E**:0-30 s) readings and the area under the curve from (**B**) 100-180 s or (**C, E**) 30-100 s was used as a measure of hP2X4- or hP2Y-mediated Ca^{2+} flux, respectively. (D) Data was normalised to the maximum ATP response in each experiment. Data presented as (**B, C, E**) mean or (**D**) mean ± SEM. (**B**) *n* = 4 - 7 and (**C-E**) *n* = 4 independent experiments.
